# Effect of Ultrasonic Pretreatment on Biomethane Potential of Two-Phase Olive Mill Solid Waste: Kinetic Approach and Process Performance

**DOI:** 10.1155/2014/648624

**Published:** 2014-08-12

**Authors:** B. Rincón, L. Bujalance, F. G. Fermoso, A. Martín, R. Borja

**Affiliations:** ^1^Instituto de la Grasa (CSIC), Avenida Padre García Tejero, 4.41012 Sevilla, Spain; ^2^Departamento de Ingeniería Química y Química Inorgánica, Facultad de Ciencias, Universidad de Córdoba, Campus Universitario de Rabanales, Edificio C-3, Carretera Madrid-Cádiz, km 396, 14071 Córdoba, Spain

## Abstract

The effect of ultrasound (US) pretreatment on two-phase olive mil solid waste (OMSW) composition and subsequent anaerobic biodegradation was evaluated by chemical oxygen demand solubilization and biochemical methane potential (BMP) tests. OMSW was ultrasonically pretreated at a power of 200 W and frequency of 24 kHz for time periods of 20, 40, 60, 90, 120, and 180 minutes, corresponding to specific energies of 11367, 21121, 34072, 51284, 68557, and 106003 kJ/kg total solids, respectively. In order to evaluate the US pretreatment, a low, medium, and high exposure time, that is, 20, 90, and 180 min, were selected for BMP tests. Methane yields of 311 ± 15, 393 ± 14, and 370 ± 20 mL CH_4_/g VS_added_ (VS: volatile solids) were obtained for 20, 90, and 180 minutes, respectively, while the untreated OMSW gave 373 ± 4 mL CH_4_/g VS_added_. From a kinetic point of view, the BMP tests showed a first exponential stage and a second sigmoidal stage. In the first stage, the kinetic constant obtained for US pretreated OMSW at 20 minutes was 46% higher than those achieved for the pretreated OMSW at 90 and 180 minutes and 48% higher than that for untreated OMSW. The maximum methane production rate achieved was 12% higher than that obtained for untreated OMSW.

## 1. Introduction

The two-phase olive mill solid waste (OMSW) is the main waste produced after primary centrifugation in the two-phase olive oil mills. In the two-phase olive oil manufacturing process a horizontally mounted centrifuge is used for primary separation of the olive oil fraction from the vegetable solid material and vegetation water. The resultant olive oil is further washed to remove residual impurities before finally being separated from this wash water in a vertical centrifuge. Therefore, the two-phase olive mills produce three waste streams: wash waters from the initial cleaning of the fruit, an aqueous solid residue called OMSW, and the wash waters generated during the purification of the virgin olive oil [1]. Two-phase OMSW is the main waste produced and has a high organic matter concentration. It is also a very wet waste (60–70% humidity), containing 3% of olive oil and a complex structure formed mainly by lignin (42.6%), cellulose (19.4%), and hemicellulose (35.1%) [2]. These characteristics result in an elevated polluting load. In addition, the quantities of OMSW generated are very large; every year from two to four million tonnes are produced in countries like Spain. Both composition and quantity produced make two-phase OMSW an important environmental problem [3].

Anaerobic digestion of solid wastes is an attractive and established option for solid wastes treatment due to the excellent waste stabilization and high energy recovery [4–7]. The feasibility of the anaerobic digestion of the two-phase OMSW has been already shown [3, 5, 6]. Methane yield coefficients up to 0.244 L CH_4_/g COD_removed_ were reported [5].

Pretreatments to break complex structures could be a good option to increase the methane yields obtained through anaerobic digestion. Among the most studied pretreatments to improve the hydrolysis and solubilization of complex substrates prior to their anaerobic digestion stands out the use of ultrasounds [8–14]. Ultrasonic pretreatment consists of the application of cyclic sound pressure with a variable frequency to some wastes to disintegrate rigid structures and complex compounds [9, 10]. The chemistry of sonication as a pretreatment tool is quite complex and consists of a combination of shearing, chemical reactions with radicals, pyrolysis, and combustion [13]. During sonication, microbubbles are formed because of high-pressure applications to liquid, which cause violent collapses and high amounts of energy to be released into a small area. Consequently, because of extreme local conditions certain radicals (•HO, •H) can be formed [15, 16]. The radical reactions can degrade volatile compounds by pyrolysis processes taking place in microbubbles [16].

This technology or pretreatment is widely used in industrial plants for WAS in the UK, USA, and Australia achieving a reduction in the volatile solids (VS) content between 30% and 50% and an increase in the biogas production between 40% and 50% [17]. Ultrasound pretreatment has been widely studied for WAS with interesting results and also for other substrates: sewage sludge [9], pulp mill wastewaters [10], hog manure [11], sludge from the pulp, and paper industry [12]. The main target of the ultrasound pretreatment is to disrupt flocks and break the cellular walls making easier the access to the intracellular material for its subsequent degradation. One of the main advantages of the ultrasound pretreatment is that the use of external chemical agents is prevented and, therefore, an increase in the effluent volume is avoided [13].

The effect of the ultrasonication pretreatment for different substrates treated subsequently by anaerobic digestion has been studied during the last years due to an increase in the biogas production and a reduction in the hydraulic retention times needed [8]. Mechanisms of ultrasonic treatment are influenced by four main factors: specific energy, ultrasonic frequency, application time, and the characteristics of the substrate. The increased percentage in biogas production as well as the methane content in the biogas of a sonicated sludge usually increases with the sonication time applied [15, 16].

The increase in specific methane yield is mainly due to the increase in the net surface area of the particles and solubilization of complex organic matter [13]. The increment of the sonication time can reduce the particle size of a substrate [18], but, for very high times of exposition to the ultrasound, the effect of particle size reduction might be stopped and the opposite effect is produced [19]. Initially the flocks are broken, but at high exposure times the intracellular polymeric compounds released might favour a reflocculation process [19, 20]. This might result in negative effect for long exposure times.

The objective of this study was to evaluate the COD solubilization owing to the ultrasonication pretreatment of the two-phase OMSW at different specific energies and application times and to study the influence of this pretreatment on the methane production through biochemical methane potential (BMP) tests. A kinetic study of the different stages in the methane production was also carried out. There are no previous studies in the literature about ultrasound pretreatment of this substrate before its anaerobic digestion process.

## 2. Materials and Methods

### 2.1. Two-Phase OMSW

The two-phase OMSW used for the experiments was collected from the Experimental Olive Oil Factory located in the “Instituto de la Grasa (CSIC)” of Sevilla, Spain. OMSW was sieved through a 2 mm mesh to remove olive stone pieces; all results are presented for sieved OMSW. The olive variety used was “Lechín” from Sevilla. The main characteristics and composition of the two-phase OMSW are presented in Table 1.

### 2.2. Ultrasound Pretreatment

Ultrasound pretreatment of two-phase OMSW was performed using ultrasonication equipment Hielscher UP200S (sonotrode Micro tip 7). A maximum power of 200 W (100% amplitude), constant working frequency of 24 kHz, and a constant ultrasound intensity of 5.3 W/cm^2^ [21] were used. The ultrasound tip was used in open 100 mL Pyrex glass beakers. Ultrasound pretreatment times of 20, 40, 60, 90, 120, and 180 minutes were studied corresponding to six different specific energies and ultrasound densities [15] (Table 2). All the ultrasound pretreatment experiments were carried out in duplicate and the final results expressed as means.

Two-phase OMSW at 80% (80 g two-phase OMSW: 20 g water) was used for all the experiments with ultrasound pretreatment and without pretreatment. Temperature was not controlled during the ultrasound pretreatment. After ultrasound pretreatment, the samples were cooled to ambient temperature.

### 2.3. Biochemical Methane Potential (BMP) Tests

To compare methane yields after the pretreatment, BMP tests were used. BMP tests were carried out in reactors with an effective volume of 250 mL. Reactors were continuously stirred at 500 rpm and placed in a thermostatic water bath at mesophilic temperature (35 ± 2°C).

The reactors were sealed and the headspace of each flask was filled with nitrogen at the beginning of each assay. The methane produced was measured by liquid displacement passing the biogas through a 3N NaOH solution to capture CO_2_ assuming that the remaining gas was methane. The anaerobic digestion experiments were run for a period of 20 days until the accumulated gas production remained essentially unchanged; that is, on the last day production was lower than 2% of the accumulated methane produced. Each experiment was carried out in duplicate.

The inoculum used in the BMP assays was obtained from an industrial anaerobic reactor treating brewery wastewater and operating at mesophilic temperature. The characteristics of the anaerobic inoculum used were pH: 7.5 and VS: 22 g/L.

The inoculum to substrate ratio used was 2 on VS basis. For each flask containing 239 mL of inoculum (with a final concentration of 21 g VS/L), the amount of untreated OMSW or ultrasound pretreated OMSW needed to give the required inoculum : substrate ratio was then added to each test digester. A volume of 0.239 mL of trace element solution was also added to each digester.

The composition of the trace elements solution was FeCl_2_ *·*4H_2_O, 2000 mg/L; CoCl_2_ *·*6H_2_O, 2000 mg/L; MnCl_2_ *·*4H_2_O, 500 mg/L; AlCl_3_ *·*6H_2_O, 90 mg/L; (NH_4_)_6_Mo_7_O_24_ *·*4H_2_O, 50 mg/L; H_3_BO_3_, 50 mg/L; ZnCl_2_, 50 mg/L; CuCl_2_ *·*2H_2_O, 38 mg/L; NiCl_2_ *·*6H_2_O, 50 mg/L; Na_2_SeO_3_ *·*5H_2_O, 194 mg/L; and EDTA, 1000 mg/L. Two reactors with anaerobic inoculum and trace elements solution but without substrate addition were used as controls.

### 2.4. Analytical Methods

TS and VS were determined, according to the standard methods 2540B and 2540E, respectively [22]. Total chemical oxygen demand (CODt) was determined as described by Rincón et al. [4], while soluble chemical oxygen demand (CODs) was determined using the closed digestion and the colorimetric standard method 5220D [22]. pH was analysed using a pH-meter model Crison 20 Basic. Total alkalinity (TA) was determined by pH titration to 4.3 [22]. Hemicellulose, cellulose, and lignin were determined according to Van Soest et al. method [23]. Total Kjeldahl nitrogen (TKN) was analysed using a method based on the 4500-N_org_ B of standard methods [22]. Ammoniacal nitrogen was determined by distillation and titration according to the standard method 4500-NH_3_ E [22]. Fat was analyzed by the official method of the EEC number 2568/91 (European Community Official Diary, L248/1 of 05.09.1991). All the analyses were carried out in triplicate.

## 3. Results and Discussion

### 3.1. Influence of Ultrasound Pretreatment on the Characteristics of Two-Phase OMSW

Table 3 shows the characteristics of the two-phase OMSW after the different ultrasound pretreatments in terms of humidity, TS, VS, CODt, CODs, and COD solubilization. The degree of COD solubilization was calculated from the data of CODs measured after each pretreatment condition tested and CODt initial of the OMSW using the following equation [24, 25]:

(1) $$\begin{array}{c} \text{COD}\;\;\text{solubilization}\;\;\left(\%\right)=\left(\frac{\text{CODs}}{\text{CODt}}\right)*100. \end{array}$$

Although COD solubilizationdid not show a big variation for the chosen US exposition times (Table 3), the best solubilization levels were achieved for the pretreatments at 90 and 120 min with 57% of solubilization, followed by the treatment at 20 min and 60 min with 56% and 55% of COD solubilization, respectively (Table 3). From the low times assayed (20, 40, and 60 min), 20 min was the chosen time for the BMP tests, as for 40 and 60 min similar solubilizations were virtually achieved. For the same reason from the highest times studied (90, 120, and 180 min) the pretreatment at 90 min was selected. The pretreatment at 180 min was also assayed through BMP to compare the effect of a high exposure to ultrasound pretreatment. Therefore, the BMP tests were assayed for low, that is, 20 min, medium, that is, 90 min, and high, that is, 180 min, exposition times.

Wang et al. [26] found for WAS that the concentration of soluble COD increased with the increase in the sonication time owing to the breakage to the flocks and the disrupting of cell walls in bacteria that released the extracellular organic compounds. Shimizu et al. [8] also evaluated the solubilization of WAS at different sonication times; they found that a minimum of 30–40 min of ultrasonication time was necessary to achieve 50% of solubilization. The efficiency of ultrasonication as a pretreatment method for hog manure and WAS prior to their anaerobic digestion has been recently evaluated at specific energies of 250–30,000 kJ/kg TS [11]. The latter study confirmed that COD solubilisation from WAS correlated well with the more labour and time intensive degree of disintegration test. Hog manure was found to be more amenable to ultrasonication than WAS, as it took only 3000 kJ/kg TS to cause 15% more solubilisation as compared to 25,000 kJ/kg TS for WAS [11].

For all the tested times in the present study (20, 40, 60, 90, 120, and 180 minutes), COD solubilization slightly increased compared to the untreated sample, being for the longest time applied (180 min) and for the time of 40 min the lowest COD solubilization increase. Ultrasound pretreatment can affect the particle size; some studies establish a relationship between the increase in the sonication time and the particle size concluding that at higher exposure times higher solubilization and lower particle sizes were found, but for very long times of exposure to the ultrasound the opposite effect might be produced owing to the formation of recalcitrant compounds [9]. It has been also reported in the literature that high specific energies may induce the reagglomeration of particles, thereby shifting particle size toward higher diameters, decreasing slightly or keeping constant the solubilization levels [27]. The latter study revealed how the percentage of COD solubilization was maintained around 8% when the specific energy applied increased from 76.5 to 128.9 MJ/kg during the US pretreatment of algal biomass [27].

Other authors found that although sonication disrupted cellular matter providing a higher solubilization than without pretreatment for WAS, the solubilization resulted in soluble nonbiodegradable compounds [10]. The increase in sonication time causes more release of intracellular polymers; these biopolymers released were thought to be the glue that holds bioflocs together [13, 20].

Table 4 shows the hemicellulose, cellulose, and lignin contents of the ultrasound pretreated two-phase OMSWs compared to untreated OMSW. The highest increase in the hemicellulose content (31%) was obtained for the OMSW pretreated during 90 min. The increase in the cellulose content was evident for all the ultrasound pretreatments: 150%, 176%, and 162% for 20, 90, and 180 min, respectively, compared to the untreated two-phase OMSW. In the same way, an increase of 54% in the percentage of cellulose with respect to its initial content in the substrate was observed in the sunflower oil cake after sonication with a specific energy of 24.000 kJ/kg TS [28].

### 3.2. Impact of Ultrasound Pretreatment on Biochemical Methane Potential

**Figure 1:**
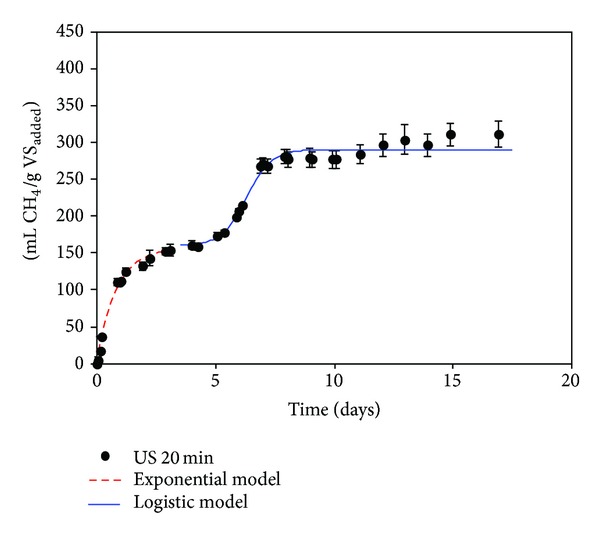
Cumulative methane yield, expressed as mL CH_4_/g VS_added_, obtained during the BMP tests carried out with pretreated OMSW with ultrasound during 20 minutes.

**Figure 2:**
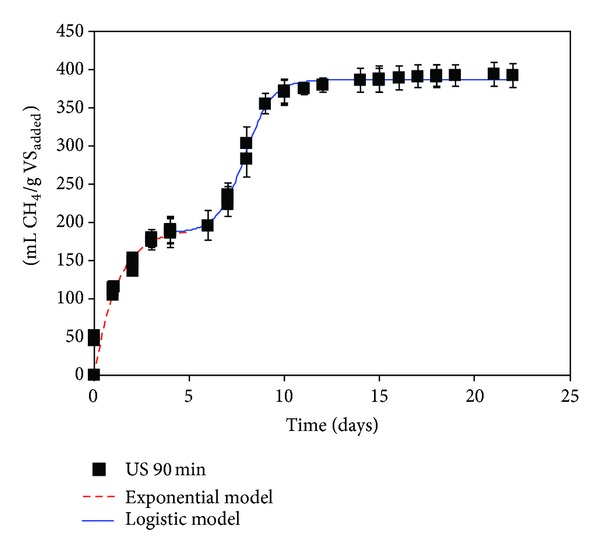
Cumulative methane yield, expressed as mL CH_4_/g VS_added_, obtained during the BMP tests carried out with pretreated OMSW with ultrasound during 90 minutes.

**Figure 3:**
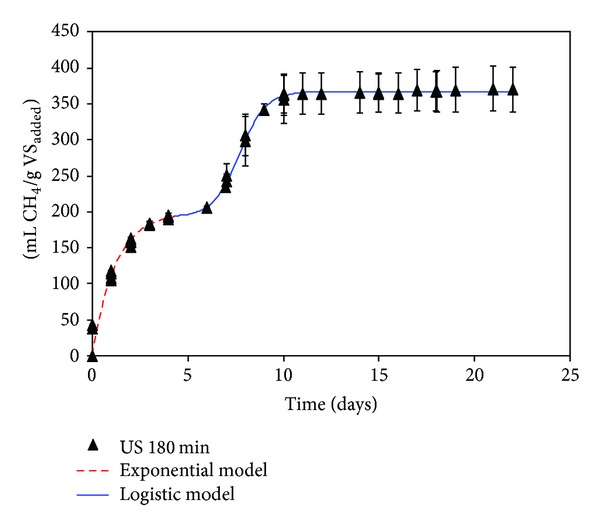
Cumulative methane yield, expressed as mL CH_4_/g VS_added_, obtained during the BMP tests carried out with pretreated OMSW with ultrasound during 180 minutes.

**Figure 4:**
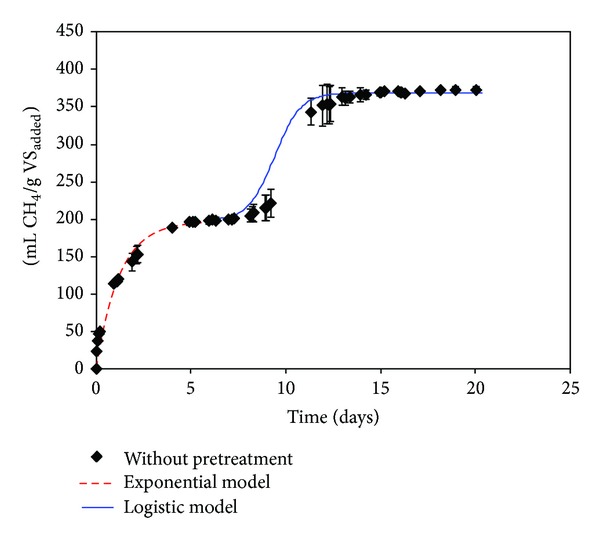
Cumulative methane yield, expressed as mL CH_4_/g VS_added_, obtained during the BMP tests carried out with untreated OMSW.

The methane yields obtained through BMP after 20 days of digestion for the ultrasound pretreatments selected were 311 ± 15, 393 ± 14, and 370 ± 20 mL CH_4_/g VS_added_ for pretreated OMSW during 20, 90, and 180 min, respectively (Figures 1–3), and 373 ± 4 mL CH_4_/g VS_added_ for OMSW without ultrasound pretreatment (Figure 4). The maximum value of methane yield was obtained after a pretreatment time of 90 minutes with a specific energy of 51284 kJ/kg TS and this maximum value was only 5.6% higher than that obtained for untreated OMSW. Higher increments in biogas production and methane yields were reported after sonication of other substrates when compared with untreated samples. For instance, Bougrier et al. [29] showed an increase in the methane yield of WAS from 221 to 334 mL CH_4_/g COD_added_ after an ultrasonic pretreatment at 9350 kJ/kg TS, which was more effective than other pretreatments assessed such as ozonation or thermal pretreatment. In the same way, an increase in the methane production of 44% was also reported by Erden and Filibeli [30] for WAS previously sonicated with a specific energy of 9690 kJ/kg TS. Likewise, an improvement of 16% in specific biogas production was also observed after ultrasonic pretreatment of WAS with a high content of polycyclic aromatic hydrocarbons at a specific energy of 11000 kJ/kg TS [16]. Similarly, the methane potential of hog manure increased by 20.7% in comparison with unsonicated manure for a specific energy input of 30000 kJ/kg TS [11], which is lower than that used in the present work for obtaining the maximum methane yield (51284 kJ/kg TS).

In the present study, methane yield slightly increased when the pretreatment time was increased from 20 to 90 minutes and the specific energy consequently increased from 11367 to 51284 kJ/kg TS. A slight decrease in the methane yield was observed for an exposure time of 180 minutes with a specific energy of 106003 kJ/kg TS. The methane yield increase from 20 to 90 minutes may be attributed to the transformation of the particulate part of the substrate to more soluble substances by ultrasonication [28]. When high times of exposition (e.g., 180 min.) are applied the opposite effect can be produced. The organic structures may get more complex owing to the polymeric matter released [13], becoming more difficult to biodegrade. Moreover, high intensive degree times of disintegration test have been found responsible for refractory compound formation and generation of soluble nonbiodegradable compounds which can inhibit methane production [11].

It has been recently demonstrated that the increased solubilization provoked by thermal and ultrasonic pretreatments on mixed-microalgal biomass was not followed by an increased methane production in BMP tests [31]. In the latter study the pretreatments enhanced the transformation of simple sugars to smaller carbon organic acids, especially propionic acid, which results in inhibition of methanogenic microorganisms at certain concentrations [31]. Alzate et al. [32] have recently demonstrated the lack of correlation between the solubilization degree and methane enhancement potential in BMP tests of microalgae mixtures subjected to ultrasound pretreatment. They found no increases in methane productivity with increases in energy inputs at applied energies higher than 10.000 kJ/kg TS.

### 3.3. Effect of Ultrasound Pretreatment on the Process Kinetics

Figures 1, 2, 3, and 4 show the evolution of methane production with time for ultrasonically pretreated two-phase OMSW at 20, 90, and 180 min and untreated OMSW, respectively.

Two different stages were observed for all the cases studied: a first stage during the first 5–7 days of operation followed by an intermediate adaptation period or lag stage and finally a second stage, in which the methane production rate increased gradually to become almost zero at the 20–25 days of digestion.

In order to simulate the two stages observed, two different models were used and selected as previously by Rincόn et al. [4] with thermally pretreated OMSW: a first-order exponential model for the first stage which is commonly applicable to easily biodegradable substrates [33] and a second sigmoidal or logistic model with its three characteristic phases, that is, lag, exponential increase, and final stabilization step [34].

#### 3.3.1. First Phase: First-Order Exponential Model

The first-order exponential model is given by the following expression:

(2) $$\begin{array}{c} B_{1}=B_{\max}\cdot \left[1-\exp\left(-K\cdot t\right)\right], \end{array}$$

where *B*_1_ (mL CH_4_/g VS_added_) is the cumulative specific methane production, *B*_max⁡_ (mL CH_4_/g VS_added_) is the ultimate methane production, *K* is the specific rate constant or apparent kinetic constant (days^−1^), and *t* (days) is the time.

This model was applied for the first experimental stage of methane production or exponential step (from 0 to 5–7 days) for all the substrates tested. The adjustment by nonlinear regression of the pairs of experimental data (*B*_1_, *t*) using the Sigmaplot software (version 11.0) allowed the calculation of the parameters *K* and *B*_max⁡_ for this first stage of methane production (Table 5). The high values of the *R* ^2^ and the low values of the standard error of estimate (S.E.E.) for the cases tested demonstrate the goodness of the fit of experimental data to the model proposed for this first exponential stage.

Table 5 shows the specific rate constants (*K*) obtained for the first stage of digestion (untreated two-phase OMSW and pretreated two-phase OMSWs at 200 W during 20, 90, and 180 min) with values ranging between 0.82 ± 0.06 and 1.21 ± 0.14 days^−1^. *K* was significantly higher for the ultrasound pretreatment at 20 min (*K* = 1.21 d^−1^) than for the other times studied. For the other pretreatment times studied, that is, 90 and 180 min, and for the untreated OMSW, the *K* values were practically similar ranging between 0.82 and 0.83 d^−1^. Therefore, the kinetic constant for the ultrasound pretreatment at 20 minutes was 46% higher than those obtained for the pretreated OMSW at 90 and 180 minutes and 48% higher than for untreated OMSW. The highest value of *K* (1.21 days^−1^) achieved for the pretreated OMSWs during 20 min might be associated with its lower lignin (14.4%) and hemicellulose concentrations (10.9%) after pretreatment compared to the other pretreatment conditions (Table 4). In addition, the values of the kinetic constants obtained in the present research work for the ultrasound pretreated OMSW at 90 and 180 minutes were of the same order of magnitude as those obtained in BMP tests of thermally treated OMSW at 180°C during 180 minutes [4].

During the first stage the ultimate methane production, *B*_max⁡_, for the US (20 min) was somewhat lower (158 mL CH_4_/g VS_added_) than those obtained for the other pretreatment times, whose value ranged between 191 mL CH_4_/g VS_added_ (US 90 min) and 199 mL CH_4_/g VS_added_ (US 180 min). These results might indicate a slight increase of easily degradable compounds after 90 and 180 minutes of pretreatment but still with a high percentage of complex substrates diminishing the degradation rate.

#### 3.3.2. Second Phase: Sigmoidal or Logistic Model Application

For the second stage of methane production, that is, between the 5th and 7th days and last day of the operating period, 25th day, the following logistic model (3) was used to estimate process performance [4, 33, 34]:

(3) $$\begin{array}{c} B_{2}\;=\;B_{0}\;+\;\frac{P}{[1\;+\exp\left(-4\cdot R_{m}\cdot \left(t-\lambda \right)/\left(P\;+\;2\right)\right)]}, \end{array}$$

where *B*_2_ is the cumulative methane production during the second stage (mL CH_4_/g VS_added_), *B*_0_ is the cumulative methane production at the startup of the second stage (mL CH_4_/g VS_added_) and should approximately coincide with the value of *B*_max⁡_ obtained at the end of the first stage,* P* is the maximum methane production obtained in the second stage (mL CH_4_/g VS_added_), *R*_*m*_ is the maximum methane production rate (mL CH_4_/g VS_added_ d), and *λ* is the lag time (days).

The logistic model assumes the rate of methane production to be proportional to microbial activity [35]. This model has been previously used for estimating the methane production in batch anaerobic digestion experiments of different substrates such as landfill leachate, herbaceous grass materials, and sewage sludge [33–36].

For the logistic model the maximum methane production obtained in the second stage (*P*) had the maximum value for the 90-minute pretreatment (200 mL CH_4_/g VS_added_) followed by the 180-minute pretreatment (174 mL CH_4_/g VS_added_), untreated OMSW (171 mL CH_4_/g VS_added_), and pretreatment during 20 minutes (130 mL CH_4_/g VS_added_) (Table 6). Moreover, comparing the values of the *R*_*m*_ or maximum methane production rates obtained in the logistic model (Table 6) the best pretreatment was the US (90 min). For the ultrasound pretreatment at 90 min the kinetics was the quickest; 70.5 mL CH_4_/(g VS_added_ *·*day) was produced, a value 12% higher than that obtained for untreated OMSW and 9.5% and 10.3% higher than that obtained at 20 and 180 minutes, respectively.

The ultrasound pretreatment during 90 minutes most likely promotes the release of more easily biodegradable compounds, which allowed an increase in the *R*_*m*_ and a decrease in the lag period.

The shortest lag phase (*λ*) was obtained for US (20 min), that is, 6.3 days, while the longest lag phase was achieved for the untreated OMSW, that is, 9.4 days. Long lag phases can lead to the generation of different inhibitor compounds that delay the startup of the second phase in the methane production [34]. The lowest *R*_*m*_ value, that is, 62.7 mL CH_4_/g VS_added_ *·*d, was obtained for the pretreatment with the highest lag phase, that is, 9.4 days (untreated OMSW). This value of *R*_*m*_ was very similar to that achieved in BMP tests of OMSW previously treated thermally at 180°C during 180 min [4].

**Figure 5:**
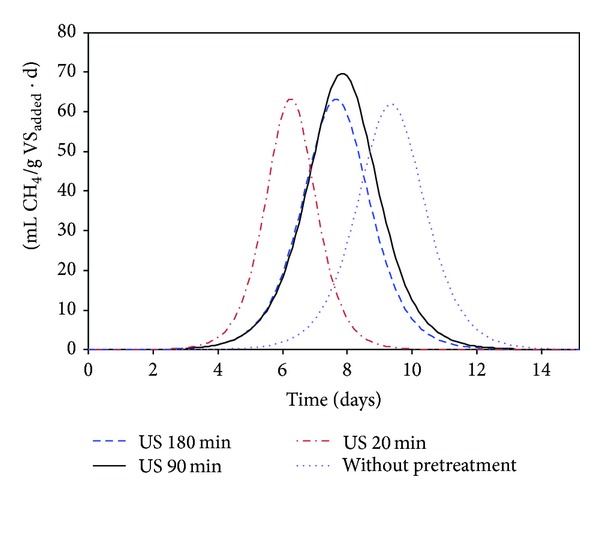
Methane production rate, expressed as mL CH_4_/(g VS d), obtained during the second stage of the BMP tests carried out with untreated OMSW and ultrasound pretreated OMSW during 20, 90, and 180 minutes.

The first derived *B*_2_ with respect to the digestion time gives the evolution of the methane production rate with time during the second stage (mL CH_4_/(g VS*·*day)) (Figure 5). The degradation rate of ultrasound pretreated OMSW during 90 minutes was the fastest of the four conditions tested, achieving a maximum methane production rate (*R*_*m*_) of 70.5 mL CH_4_/(g VS*·*day) after 7.9 days of digestion period. Although the maximum methane production rate for ultrasound pretreated OMSW for 20 minutes was somewhat lower (64.4 mL CH_4_/(g VS*·*day)) than that mentioned for pretreated OMSW during 90 minutes, it was achieved at a lower time of 6.3 days. Finally, the methane production rate for untreated OMSW achieved the lowest *R*_*m*_ value and it needed the highest time (9.4 days) to be reached.

Ultrasound pretreatment during 90 minutes gives the most promising results for a fast OMSW degradation process making available a large concentration of soluble and biodegradable components. Ultrasound pretreatment during 180 minutes had opposite effect, indicating a possible recalcitrant compound formation [13, 20].

### 3.4. Energy Balance

A net balance of the consumed energy in the pretreatment and the produced energy through BMP for the ultrasound pretreated OMSW was found to be negative for all pretreatment times and specific energies studied. The less unfavorable energy balance was observed for the lowest exposure time and specific energy studied, that is, 20 minutes, with a negative energy balance of −1830 kJ/kg TS. For pretreatments of 90 and 180 minutes the energy balance between the consumed energy in the pretreatment (input energy) and produced energy through anaerobic digestion (output energy) was obviously more negative.

A similar negative energy balance has been recently reported in the evaluation of ultrasonic pretreatment combined with anaerobic digestion of mixed-microalgal biomass [31]. After applying thermal, ultrasonic, and alkali pretreatments to raw microalgae biomass to promote the anaerobic digestion efficiency through BMP tests it was observed that only the chemical pretreatment yielded slightly higher energy gains than that of nonpretreatment condition, while the energy balance with the ultrasonic pretreatment gave a negative value of −220 kJ/kg VS using a pretreatment time Of 180 seconds [31]. Houtmeyers et al. [37] reported that the pretreatment of WAS with ultrasounds and microwave both with energy specific of 2100 kJ/kg sludge was economically not feasible although an increase in the biogas production of 27% (microwave pretreated) and 23% (ultrasonic pretreated) was observed with respect to untreated samples. Likewise, a study of the effect of ultrasonic pretreatment on methane production potential from some corn ethanol products (distiller's wet grains, thin stillage, and condensed distiller's solubles) revealed that ultrasonic pretreatment required more energy than was generated by the process in terms of additional biogas production giving a negative energy balance [38]. The efficiency and economic viability of ultrasonication as a pretreatment method for hog manure anaerobic digestion was evaluated at specific energies of 250–30000 kJ/kg TS [11]. Hog manure was found more amenable to ultrasonication than waste activated sludge, as it took only 3000 kJ/kg TS to cause 15% more solubilization as compared to 25000 kJ/kg TS for waste activated sludge. It was noted in this study that biomass cell rupture occurred at specific energy of 500 kJ/kg TS. However, an economic evaluation indicated that only a specific energy of 500 kJ/kg TS was economical, with a net energy output valued at $4.1/ton of dry solids, due to a 28% increase in methane production [11].

## 4. Conclusions

Ultrasound pretreatment of two-phase OMSW at a power of 200 W (100% amplitude) and a constant frequency of 24 kHz during 20, 90, and 180 minutes increased the COD solubilization of this substrate compared to the untreated sample. The best methane yield obtained through BMP tests, 393 mL CH_4_/g VS_added_, was achieved for ultrasound pretreatment during 90 min; this yield was 5.6% higher than that obtained for OMSW without pretreatment. Moreover, taking into account the kinetics of the two stages observed during methane production (exponential and sigmoidal curves), the highest maximum methane production rate, *R*_*m*_, was also achieved for ultrasound pretreatment during 90 min. The maximum value of  *R*_*m*_ was found for ultrasound pretreatment during 90 min, values 12%, 9.5%, and 10.3% higher than that obtained for untreated OMSW and OMSW pretreated at 20 and 180 min, respectively. A net balance between the consumed energy during the pretreatment and energy production through BMP gave a negative value for all the cases studied.

## Figures and Tables

**Table 1:** Main characteristics and composition of the two-phase OMSW used in the experiments.

Parameters	Values
TS (g/kg)	265 ± 3
VS (g/kg)	228 ± 2
CODt (g O_2_/kg)	331 ± 1
CODs (g O_2_/kg)	143 ± 3
Ph	4.9 ± 0.2
TA (g CaCO_3_/kg)	2.5 ± 0.1
AN (g ammoniacal N/kg)	0.3 ± 0.0
TKN (g Kjeldahl N/Kg)	3.6 ± 0.1
Hemicellulose (%)	11.3 ± 0.2
Cellulose (%)	5.2 ± 0.1
Lignin (%)	19.7 ± 0.4
Fat (%)	3.8 ± 0.3

TS: total solids; VS: volatile solids; CODt: total chemical oxygen demand; CODs: soluble chemical oxygen demand; TKN: total Kjeldahl nitrogen; AN: ammoniacal nitrogen; TA: total alkalinity.

**Table 2:** Experimental conditions of specific energies and ultrasound densities applied to the two-phase OMSW used in the experiments (80% w/w).

Time	Wet sample	Specific energy	Ultrasound density
(min)	(g)	(kJ/kgTS)	(W/kg wet sample)
20	99.6	11367	2008.2
40	107.2	21121	1865.8
60	99.7	34072	2006.6
90	99.3	51284	2013.5
120	99.1	68557	2018.8
180	96.1	106003	2080.9

**Table 3:** Characteristics of the two-phase OMSW used (80% w/w) after different ultrasound pretreatment times and without pretreatment.

Time	Moisture	TS	VS	CODt	CODs	Solubilization
(min)	(%)	(g/kg)	(g/kg)	(g O_2_/kg)	(g O_2_/kg)	(%)
Untreated OMSW	78.8 ± 0.2	212.0 ± 2.6	182.7 ± 2.3	265.4 ± 0.7	114.7 ± 3.2	43
20	79.9 ± 0.2	206.4 ± 0.2	177.7 ± 1.9	331.3 ± 1.0	148.3 ± 0.2	**56**
40	80.8 ± 0.1	191.8 ± 0.9	164.1 ± 0.2	331.4 ± 0.0	130.9 ± 0.0	49
60	78.6 ± 0.4	213.8 ± 3.6	181.4 ± 5	376.6 ± 0.4	146.8 ± 0.0	55
90	78.3 ± 0.3	217.3 ± 2.9	183.8 ± 0.0	370.0 ± 0.1	151.0 ± 0.0	**57**
120	79.3 ± 0.3	206.8 ± 3.2	173.3 ± 0.0	385.2 ± 0.6	150.6 ± 0.1	57
180	77.9 ± 0.5	221.4 ± 5	188.6 ± 7.4	377.5 ± 0.0	126.1 ± 0.0	**48**

TS: total solids; VS: volatile solids; CODt: total chemical oxygen demand; CODs: soluble chemical oxygen demand.

**Table 4:** Hemicellulose, cellulose, and lignin contents for the untreated two-phase OMSW and ultrasound pretreated OMSW at 20, 90, and 180 min.

Times	Hemicellulose	Cellulose	Lignin
(min)	(%)	(%)	(%)
Untreated OMSW	9.0 ± 0.2	4.2 ± 0.1	15.8 ± 0.4
20	10.9 ± 0.3	10.5 ± 0.5	14.4 ± 0.6
90	11.8 ± 0.0	11.6 ± 0.6	14.6 ± 0.1
180	11.6 ± 2.6	11.0 ± 1.2	14.5 ± 1.3

**Table 5:** Kinetic parameters obtained from the exponential model in the BMP tests of untreated OMSW and ultrasound pretreated OMSW at 20, 90, and 180 min.

Time	*B*_max⁡_	*K*	*R* ^2^	S.E.E.
(min)	(mL CH_4_/g VS_added_)	(days^−1^)		
Untreated OMSW	197 ± 4	0.82 ± 0.06	0.97	11.3
20	158 ± 7	1.21 ± 0.14	0.94	5.2
90	191 ± 11	0.83 ± 0.17	0.92	12.2
180	199 ±10	0.83 ± 0.16	0.93	7.7

*B*_max⁡_ is the ultimate methane production; *K* is the specific rate constant or apparent kinetic constant. Parameters from the nonlinear regression fit: *R* ^2^: coefficient of determination; S.E.E.: standard error of estimate.

**Table 6:** Kinetic parameters obtained from the logistic model in the BMP tests of untreated OMSW and ultrasound pretreated OMSW at 20, 90, and 180 min.

Time	*B*_0_	*P*	*R*_*m*_	*λ*	*R* ^2^	S.E.E.
(min)	(mL CH_4_/g VS_added_)	(mL CH_4_/g VS_added_)	(mL CH_4_/g VS*·*d)	(days)		
Untreated OMSW	198 ± 4	171 ± 4	62.7	9.4 ± 0.1	0.99	3.4
20	160 ± 5	130 ± 55	64.4	6.3 ± 0.8	0.96	7.6
90	187 ± 9	200 ± 9	70.5	7.9 ± 0.2	0.99	5.3
180	193 ± 8	174 ± 8	63.9	7.7 ± 0.2	0.99	2.1

*B*_0_ is the cumulative methane production at the startup of the second stage, *P* is the maximum methane production obtained in the second stage, *R*_*m*_ is the maximum methane production rate, and *λ* is the lag time. Parameters from the nonlinear regression fit: *R* ^2^: coefficient of determination; S.E.E.: standard error of estimate.
